# Role of HDAC6 and Its Selective Inhibitors in Gastrointestinal Cancer

**DOI:** 10.3389/fcell.2021.719390

**Published:** 2021-12-02

**Authors:** Bingyi Zhou, Deliang Liu, Yuyong Tan

**Affiliations:** ^1^ Department of Gastroenterology, The Second Xiangya Hospital, Central South University, Changsha, China; ^2^ Research Center of Digestive Disease, Central South University, Changsha, China

**Keywords:** histone deacetylases, histone deacetylase inhibitor, gastrointestinal cancer, deacetylation, ubiquitination

## Abstract

Worldwide, cancer is the second leading cause of mortality after cardiovascular diseases. Among the numerous malignant tumors in human, digestive system cancers are the primary cause of morbidity and mortality. Acetylation and deacetylation are crucially involved in cancer occurrence and development; in addition, the deacetylation process is regulated by histone deacetylases (HDACs). Among the 18 human HDACs that have been reported, HDAC6 has been widely studied. There is upregulated HDAC6 expression in numerous types of tumor tissues and is closely associated with clinicopathological characteristics. Moreover, several HDAC6 inhibitors have been identified; furthermore, there has been extensive research on their ability to inhibit the growth of many tumors. This review summarizes the roles of HDAC6 in different primary digestive system malignancies.

## Introduction

Gastrointestinal cancers involve various diseases, with many having poor prognoses. The incidences of colorectal and stomach cancers remains high; moreover, there are considerable mortality rates of cancer in the esophagus, liver, pancreas, stomach, and colon (Ferlay et al., 2019). There are various clinical treatment methods for improving the survival rate of patients with gastrointestinal tumors, including surgery, endoscopic treatment, chemotherapy, and radiotherapy (Kudo, 2017; Zhou and Zhang, 2019). However, these treatment methods have unsatisfactory effects especially in tumor metastasis, with resistance to radiotherapy/chemotherapy and recurrence (DE Simone et al., 2017; Langer and Becker, 2018). Therefore, the specific mechanisms underlying gastrointestinal cancer and novel therapeutic options for these diseases must be developed.

**FIGURE 1:**
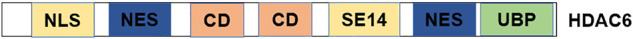
Structure of HDAC6 protein. The HDAC6 protein contains two functional catalytic domains (CD) that catalyze deacetylation. The nuclear export signal (NES) helps cytoplasmic localization, and the Ser-Clu–containing tetrapeptide (SE14) is responsible for the stable anchorage of the enzyme in the cytoplasm. Its nuclear localization sequence (NLS) mainly locates in the cytoplasm due to the interaction of NES and SE14 motifs. In the C-terminal region of the protein, the ubiquitin-binding zinc finger domain (UBP) constitutes a high-affinity ubiquitin-binding motif.

**FIGURE 2:**
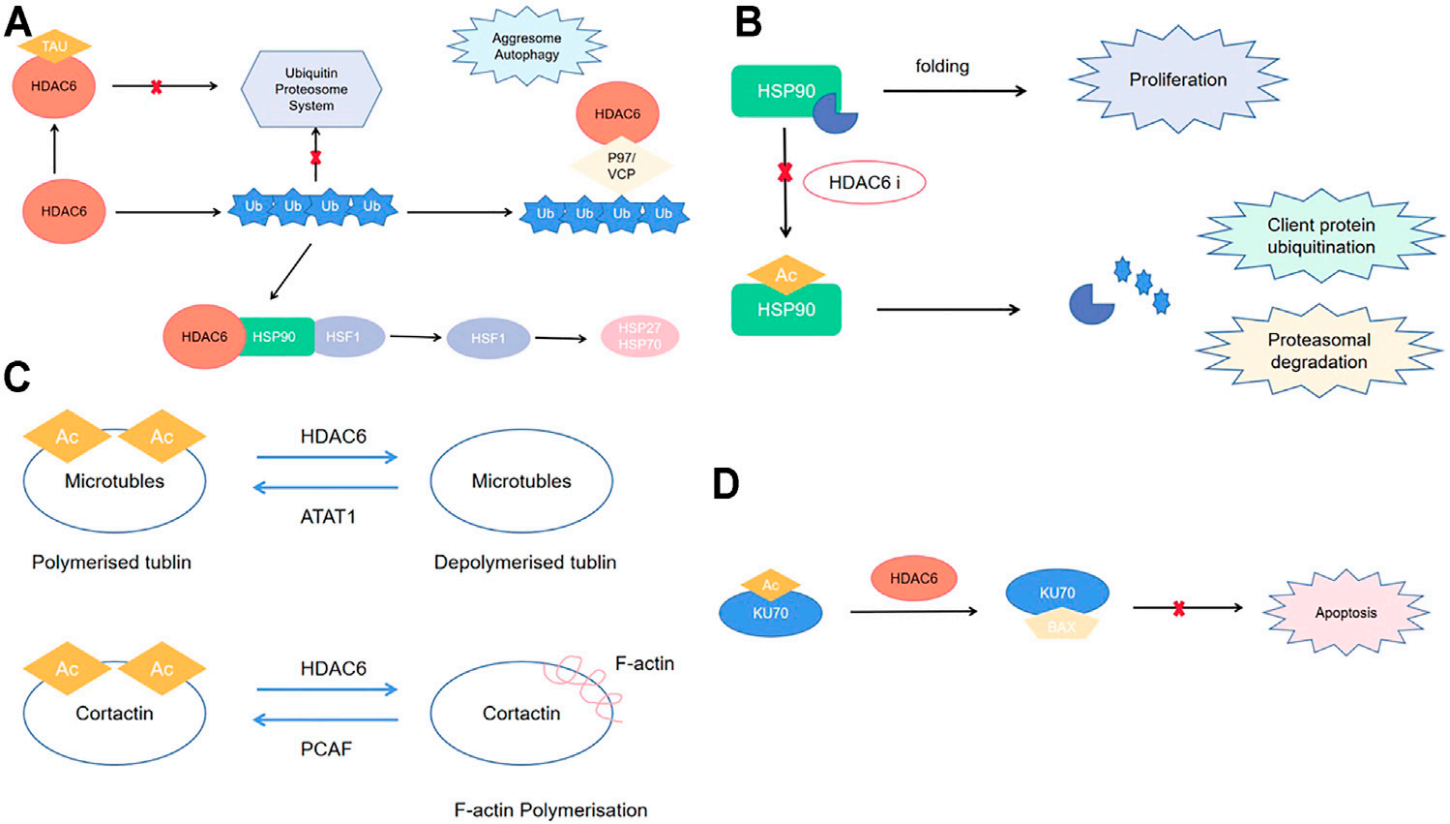
**(A)** HDAC6 role in ubiquitin proteosome system; **(B)** HDAC6 role in cell migration, invasion, adhesion, microtubule, and cytoskeletal dynamics; **(C)** HDAC6 role in proteasomal degradation; **(D)** HDAC6 role in apoptosis pathway.

Epigenetic modifications, including histone acetylation and deoxyribonucleic acid (DNA) methylation, are closely associated with tumorigenesis and cancer progression (Chen et al., 2019a; Salek Farrokhi et al., 2020). There have been recent reports of the importance of histone deacetylase (HDAC)–mediated epigenetic process in the carcinogenesis (Schizas et al., 2020; Souza and Chatterji, 2015). This posttranslational modification is reversible; furthermore, it has significant effects on chromatin structure/function and regulation of eukaryotic gene expression (Sanaei and Kavoosi, 2019). Previous studies have reported 18 human HDACs, which are divided into four classes based on their homology to yeast HDACs: classes I, II, III, and VI. Class II comprises classes IIa and IIb (de Ruijter et al., 2003). Among them, HDAC6 is the most widely studied class IIb HDAC. The HDAC6 gene is in Xp11.23 and is responsible for encoding a protein with 1,215 amino acids. There are two homologous functional catalytic domains in HDAC6, which have independent functions. The ubiquitin-binding zinc finger domain (ZnF-UBP domain) located at C-terminal end of HDAC6 is crucially involved in regulating ubiquitination-mediated degradation (Boyault et al., 2007a). Moreover, with the nuclear export sequence (NES) and the SE14 motif for cytoplasmic retention, HDAC6 is mainly localized to the cytoplasm, whereas other HDACs are localized in the nucleus (de Ruijter et al., 2003) (Figure 1). HDAC6 exerts its function through deacetylation-dependent and -independent way (Li et al., 2013; Pulya et al., 2021). For example, HDAC6 plays important role in ubiquitin proteosome system through proteosomal degradation of HSP90 (Matthias et al., 2008; Boyault et al., 2007b) (Figures 2A,B). Moreover, it regulates cell migration, invasion, adhesion, and microtubule and skeletal dynamics through deacetylation of tubulin and cortactin (Miyake et al., 2016; Zhang et al., 2003) (Figure 2C). In addition, HDAC6 involves the apoptosis pathway via deacetylation of Ku70 (Kerr et al., 2012) (Figure 2D).

Given the extensive research on the special structure, substrate, and biological function of HDAC6, it could be considered as an important potential therapeutic target for numerous diseases, including cancer (Lee et al., 2008), neurodegenerative disease (Krukowski et al., 2017), and inflammatory diseases (Yan et al., 2018). Furthermore, HDAC6 inhibitors have been widely investigated, with some being found to act effectively with antitumor agents or other drugs (Li et al., 2017; Hanikoglu et al., 2018). For example, colorectal and breast cancer cells are inhibited through treatment with a combination of thiol-based inhibitors and paclitaxel (Itoh et al., 2007). In addition, the proteasome inhibitors, bortezomib and tubacin, or acrylamide‐containing inhibitor, allow synergical prevention of cell motility and deduction of cytotoxicity in multiple cancers (Bazzaro et al., 2008; Santo et al., 2012). Although other pan-HDAC inhibitors have several adverse effects, including hematological toxicity and QT prolongation, HDAC6 inhibitors could have wider prospect for clinical use given their high selectivity (Batchu et al., 2016). This review focuses on recent studies of HDAC6 in gastrointestinal cancers, including esophageal, gastric, colorectal, liver, and pancreatic cancer, as well as cholangiocarcinoma (CCA). In addition, we briefly summarized recent studies on the inhibitors of HDAC6 in these cancers, which suggests that this versatile enzyme is an attractive therapeutic target.

## GI Cancers That HDAC6 Serves as an Oncogene

### Esophageal Cancer

Esophageal cancer is a common, highly invasive malignant tumor of the digestive tract (Pennathur et al., 2013), Moreover, it ranks seventh and sixth in terms of incidence and mortality, respectively (Bray et al., 2018). Esophageal squamous cell carcinoma (ESCC) accounts for approximately 80% of esophageal cancers. HDAC6 expression in ESCC and the role of HDAC6 inhibitors on ESCC have been studied.

Tao et al. (2018) reported that HDAC6 was overexpressed in ESCC cell lines (KYSE140, KYSE170, KYSE180) compared with noncarcinoma esophageal epithelial cell 1 (HEEC-1). Xie et al. (2018) reported that HDAC6 expression in metastatic lymph nodes (MLNs) is significantly decreased compared with primary tumor in T3N1-2M0 ESCC tissues. HDAC6 expression in MLNs but not primary tumor was an independent prognostic factor: patients with low expression of HDAC6 in MLNs had a better overall survival and disease-free survival. However, they did not compare HDAC6 expression levels in primary tumors, MLN of noncancerous tissue.

Regarding inhibitors, Tao et al. (2018) confirmed that HDAC6 downregulation by specific siRNA could inhibit cell proliferation and reduce cell migration by increasing α-tubulin and HSP90 acetylation. Liu et al. (2019) discovered that MiR-601 could downregulate HDAC6 expression in ESCC, which eventually suppressed proliferation, invasion, and migration of ESCC cells. Cao et al. (2018) reported that HDAC6 inhibition using the selective inhibitor ACY1215 could inhibit cellular proliferation as well as induce G2/M arrest and apoptosis *via* miR-30d/phosphoinositide 3-kinase (PI3K)/AKT/mTOR and ERK pathways. Moreover, HDAC6 may be among the targets of bortezomib tumor suppression in esophageal carcinoma cells (Ao et al., 2019). Overall, HDAC6 serves as an oncogene in esophageal cancer.

### Colorectal Cancer

Colorectal cancer (CRC) ranks third and second in cancer incidence and mortality, respectively, which makes it among the most common malignant tumors worldwide (Bray et al., 2018). Comprehensive therapy for patients with CRC, including surgery, chemotherapy, radiotherapy, and biological target therapy, significantly improves their prognosis. Nonetheless, more than half of patients with CRC die due to tumor recurrence or metastasis (Benson et al., 2017). Notably, the potential mechanism underlying CRC should be elucidated; therefore, there is a need to research CRC pathogenic mechanism or novel therapeutic method.

We previously (Zhang et al., 2019; Zhang et al., 2020) observed HDAC6 overexpression in CRC tissue, which could be an independent risk factor for poor prognosis in patients with CRC. HDAC6 knockdown reduced CRC cell viability, colony formation, and migration; furthermore, HDAC6 inhibition partially inhibited the growth and migration of CRC cells through the mitogen-activated protein kinase/extracellular signal–regulated kinase (MAPK/ERK) pathway. Moreover, SET7 inhibited cell proliferation and migration by acting on HDAC6 substrate; furthermore, it is involved in tumor suppression by increasing levels of acetylated α-tubulin mediated by HDAC6. The interaction between SET7 and HDAC6 decreases the phosphorylated ERK (p-ERK)/ERK ratio, which indicated that it may partly suppress ERK signaling pathway. Moreover, as SPOP is an upstream negative regulator of HDAC6 stability, we observed HDAC6 oncoprotein upregulation, which facilitates tumorigenesis and metastasis after the loss-of-function mutations of SPOP (Tan et al., 2017). In addition, Hu et al. (2018) reported that HDAC6 can interact with Snail 2; subsequently, HDAC6 and PRC2 are recruited to the E-cadherin promoter, which inhibits E-cadherin expression, promotes EMT, and induces CRC invasion and metastasis. Furthermore, Wang et al. (2019) showed that HDAC6 deacetylase underlies FRA1 deacetylation at the Lys-116 residue located within its DNA-binding domain, which upregulates FRA1 transcription, followed by transactivation of NANOG expression and creation of stem-like cellular features in CRC. Fang et al. (2014) conducted a comprehensive genomic analysis of metastatic colon cancer to the lung and showed that some of these mutated genes (ADCY9, ADCY2, GNB5, K-ras, HDAC6, and ARHGEF17) are crucially involved in the phospholipase C signaling network; furthermore, they may effect on the lung metastasis in colon cancer. Chen et al. (2017) observed that PKCε-dependent phosphorylation of migration and invasion inhibitory protein (MIIP) stimulated by EGF could inhibit RelA deacetylation, activate RelA transcription, and promote tumor metastasis in EGF-stimulated HCT116 cells.

Among the HDAC6 inhibitors, ACY-1215 had been widely used in clinical trials to treat hematological malignancies. In our previous study (Tan et al., 2019), ACY1215 inhibited cell proliferation, migration, and invasion and induced apoptosis in HCT116 cells. Furthermore, ACY1215 may enhance the chemotherapeutic effect of 5-fluorouracil in HCT116 cells. Lee et al. (2018) suggested that combining ACY-1215 and oxaliplatin was more potent than either drug alone in CRC cells, which caused activation of caspase-3 and poly(ADP-ribose) polymerase, increased expression of B-cell lymphoma (Bcl)-2 homologous antagonist/killer, and decreased Bcl–extra-large protein levels, p-ERK, and phosphorylated protein kinase B (pPKB) expression. Forsythe et al. (2018) reported that combining carfilzomib (proteasome inhibitor) and ACY-1215 could cause remarkable accumulation of protein aggregates, acute endoplasmic reticulum (ER) stress, apoptosis, and therapeutic efficacy in BRAFV600E mutations (BRAFMT) *in vitro* and xenograft CRC models. Further analysis revealed increased CHOP expression in the process of apoptosis, which indicates possible treatment with inducers of acute ER stress for poor prognostic BRAFMT CRC.

There are numerous known HDAC6 inhibitors for CRC. Ryu et al. (2018) compared A452 with ACY-1215 and suggested that A452 is more effective as an anticancer agent as it effectively inhibits the cell growth and viability irrespective of p53 status. Further study indicated that A452 combined with irinotecan or suberoylanilide hydroxamic acid (SAHA) is more effective than either drug alone. Aceroside VIII (Ojha et al., 2018), which is a diarylheptanoid isolated from *Betula platyphylla*, could enhance the anticancer activity of HDAC inhibitor A452 in HT29 cells. Götze et al. (2014) observed that trichostatin A (TSA) and SAHA, which are nonhistone target proteins of HDACs, resulted in a quick proteasome-dependent depletion of the Wnt transcription factor TCF7L2 by inhibiting HDAC6 and HDAC10. Lee et al. (2014) suggested that in a series of indolyl/azaindolylsulfonylcinnamic hydroxamate, compound 12, which is a 7-azaindole, showed high selectivity for HDAC6 and showed better antitumor activity than SAHA (1,N-hydroxy-N′-phenyloctanediamide) in human colorectal HCT116 cells. Moreover, Ojha et al. (2018) reported that compound 12, which is a synthesized 1-aroylindoline-hydroxamic acid, showed higher selectivity for HDAC6; moreover, it exerted significant cytotoxic effects against colorectal HCT116 cells. Cao et al. (2006) reported a novel HDAC inhibitor, CRA-026440, which causes accumulation of acetylated histone and acetylated tubulin in cultured tumor cell lines, resulting in inhibition of tumor cell growth and induction of apoptosis. They observed significantly reduced tumor growth in mice treated with HCT116 or U937 human tumor xenografts. Zhuang et al. (Yang et al., 2016) designed and synthesized a series of novel selective HDAC6 inhibitors using quinazoline as the cap. They observed that a specific compound, known as N-hydroxy-4-{2-methoxy-5-[methyl (2-methylquinazolin-4-yl)-amino]phenoxy}butanamide (23bb), could inhibit HDAC6 with high selectivity and show better tumor inhibition function than SAHA or ACY-1215. Liu et al. (2015) synthesized a series of pyrimidinedione derivatives, with compound 6,4-[5-fluoro-2,6-dioxo-3-(tetrahydro-furan-2-yl)-3,6-dihydro-2H-pyrimidin-1-ylmethyl]-N-hydroxy-benzamide showing potent antiproliferative activity as an HDAC6 inhibitor as it induces apoptosis by cleaving caspase and poly ADP-ribose polymerase (PARP). *In vivo*, compound 6 also inhibited tumor growth in CRC HCT116 cells. Kaliszczak et al. (2013) described C1A, which is a selective HDAC6 inhibitor with good pharmacokinetics and *in vivo* potency in tumor xenografts. Their subsequent study (Kaliszczak et al., 2018) showed that C1A alone or in combination with the proteasome inhibitor bortezomib could inhibit KRAS-positive CRC cell growth in response to C1A. Moreover, the combination of C1A with bortezomib induced growth of CRC xenografts. Chao et al. (2017) reported that in Caco-2 colorectal carcinoma cells, tubacin, which is an HDAC6 inhibitor, promoted the extracellular release of CD133þ tumor-derived extracellular vesicles, with accompanying downregulation of intracellular CD133. Furthermore, changes in the cellular lipid composition, absence of clonogenic ability, and decreased tendency to form multicellular aggregates. Chen et al. (2019b) reported that MPT0G612 could suppress proliferation and viability, as well as induce CRC cells apoptosis. During this process, there was autophagy activation; moreover, MPT0G612 could decrease programmed death 1 ligand expression induced by interferon γ in CRC cells. Overall, HDAC6 serves as an oncogene in CRC, and HDAC6 inhibitors can inhibit CRC progression as well as enhance the chemotherapeutic effect of other drugs.

### Pancreatic Cancer

Pancreatic adenocarcinoma is famous for its malignancy and lethality among common cancers, and there are many challenges in diagnosis and treatment (Ryan et al., 2014). Furthermore, the mortality rate has been increasing in recent years, with a poor overall 5-year survival rate of <5% and a short median survival of 6 months (Michl and Gress, 2013). The study of connection between HDAC6 and pancreatic cancer is relatively less than that of other gastrointestinal cancers, but inhibitors of other HDACs have been widely discussed (Wang et al., 2012a; Klieser et al., 2015), indicating the possibility of existed connection.

Li et al. (2014) reported higher HDAC6 expression (both protein and mRNA levels) in pancreatic cancer tissues than in normal/adjacent noncancerous tissues. However, HDAC6 lacks an obvious effect on pancreatic cancer cell proliferation or cell cycle. It impairs cell motility through interaction with cytoplasmic linker protein 170 (CLIP-170), which is primarily localized at the ends of growing microtubules. However, Wang et al. (2012a) reported no difference in HDAC6 expression levels between seven pancreatic cancer cells (BxPC-3, AsPC-1, PANC-1, CFPAC-1, MIA PaCa-2, HPAC, capan-1) and normal human pancreatic ductal epithelial cells. Niu et al. (Bae et al., 2015) examined the reciprocal loop of hypoxia-inducible factor 1α (HIF-1α)/miR-646/MIIP in pancreatic cancer and observed that MIIP could suppress the deacetylase ability of HDAC6, which promoted HIF-1α acetylation and degradation, leading to decreased HIF-1α accumulation. This study investigated the indirect mechanism through which HDAC6 affects pancreatic cancer. Activating point mutations in K-RAS have been observed in pancreatic cancer, which have a high predictive value for poor therapeutic response. Yang (Li et al., 2021) reported that HDAC6 could regulate K-RAS acetylation; moreover, HDAC6 inhibition dramatically affected the growth properties of cancer cells with activation mutants of K-RAS, which suggested the utility of therapeutic targeting HDAC6 in the treatment of pancreatic cancer with mutant forms of K-RAS. However, there is a need for a large-scale study on the direct relationship between HDAC6 expression and clinical data in patients with pancreatic cancer.

There have been several studies on inhibitors, ranging from *in vitro* to mouse model studies. Laschanzky et al. (Wood et al., 2010) reported that HDAC6 is among the main targets of pan-HDAC inhibitors. Furthermore, regarding pancreatic cancer, HDAC6 inhibition cooperates with the gemcitabine, showing strong potent anticancer effects both *in vitro* and *in vivo*, which suggests that targeting HDAC6 is a therapeutic option for pancreatic cancer. Wang et al. (2012a). reported that HDAC6 inhibition through selective inhibitors (such as tubastatin A) had only minimal effect on pancreatic cancer cell proliferation, apoptosis, cell cycle, DNA double-strand breaks, and p21 expression. However, it can enhance the antitumor effect of class I HDAC inhibitor MGCD0103. Fu et al. (2019) investigated CUDC-907, which is a novel dual-acting inhibitor of PI3K and HDAC. They found that CUDC-907 could inhibit cell proliferation in pancreatic cancer cell lines, with further studies elucidating the detailed anticancer mechanism underlying CUDC-907 in several pancreatic cell lines including Aspc-1, PANC-1, and capan-1, which inhibit the HDAC6 subunit and downregulated c-Myc protein levels. Similarly, a study using a human pancreatic cancer Aspc-1 xenograft nude mouse model observed the antitumor effect of CUDC-907 with c-Myc and Ki67 downregulation. Song et al. (2018) reported that cefoperazone sodium was a selective HDAC6 inhibitor that inhibited the migration and invasion of PANC-1 cells.

## GI Cancers in Which HDAC6 Plays Controversial Role

### Gastric Cancer

Gastric cancer (GC), which is among the most severe tumors in the digestive tract around the world, has the fifth- and third-highest prevalence and mortality rates, respectively (Bray et al., 2018). Patients with advanced GC have poor survival given the lack of early detection and precise diagnosis. Therefore, other than early detection and prognostic assessment, there is a need for more studies on new therapeutic targets for GC treatment.

Wang et al. (2012b) reported much higher HDAC6 expression in *Helicobacter pylori* (HP)–induced gastric carcinogenic tissues than in precancerous lesions, both in mRNA (by quantitative real-time polymerase chain reaction) and protein level (by immunohistochemistry). However, there have been no studies on the carcinogenic effect of HDAC6 through “loss-of-function” or “gain-of-function” experiments. Chen et al. (2021) suggested that the mRNA of HDAC6 was downregulated in GC tissue than in normal tissues using the GEPIA tool. In patients with GC, HDAC6 expression levels are associated with different Lauren classifications, clinical stages, lymph node status, treatment, and human epidermal growth factor receptor 2 status. Moreover, patients with lower HDAC6 expression levels showed longer overall survival. The above two studies reveal that HDAC6 may serve as an oncogene in GC. However, He et al. (2017) reported higher HDAC6 expression in normal and premalignant lesions than GC tissues; furthermore, decreased HDAC6 expression was associated with HP infection and TNM stage. Further studies suggested that HDAC6-positive expression was associated with better outcomes, indicating the role of HDAC6 as a tumor suppressor. Wang et al. (2021) revealed that both the mRNA and protein level of HDAC6 were increased in bile acid–induced gastric intestinal metaplasia, which is regarded as a gastric precancerous lesion, in cell model and patient specimens. Park et al. (2014) demonstrated that HDAC6 gene and protein level were upregulated in GC patients and cells, compared with noncancerous sample. In addition, they found that HDAC6 promotes malignant progression and transformation in GC through the HDAC6/rabaptin-5/epidermal growth factor receptor (EGFR) pathway. Taken together, HDAC6 might play a complex role in GC. Although it possibly might be an oncogene, further clarification is required as its expression level might be affected by HP infection status, bile acids, cell types, detected method, sample size, etc.

There are two types of HDAC6 inhibitors involved in GC. One is 2,4-ditertbutylphenol (DTBP). Song et al. (2018) reported that DTBP could induce senescence in GC cells through HDAC6 inhibition. DTBP causes mitotic catastrophe and generates multinucleated cells with an increase in polymerized tubulin. Further study demonstrated that DTBP is located at the entrance of the ligand-binding pocket of the HDAC6. The other type of HDAC6 inhibitor involved in GC is TC24. Dong et al. (2018) designed and synthesized a specific inhibitor, TC24, and found that it inhibited the proliferation and motility ability in GC cells but not normal gastric GES-1 cells. Further analysis revealed that the cytotoxic effect of TC24 in GC cells was mediated by G2/M cell cycle arrest, apoptosis, and the loss of mitochondrial membrane potential. In addition, tumor angiogenesis was suppressed by TC24 through the reduction of HIF-1α and vascular endothelial growth factor (VEGF).

### Liver Cancer

Worldwide, hepatocellular carcinoma (HCC) is the fourth most common cause of cancer-related deaths. Even with the continuous improvement of comprehensive treatment for HCC, there has been a significant annual increase in the incidence and mortality rates. (Bray et al., 2018). Among all the HDACs in HCC, HDAC6 plays a crucial role; however, the detailed mechanism underlying HDAC6 inactivation in HCC remains unclear.

Currently, the expression of HDAC6 in HCC is still controversial. Kanno et al. (2012) observed higher HDAC6 expression in three HCC cell lines than in primary cultures of hepatocytes. HDAC6 knockdown significantly downregulated the migration and invasion of all HCC cell lines. Moreover, the level of HDAC6 protein overexpression is significantly correlated with high clinical stage, tumor numbers, and invasion of vascular and intrahepatic metastases. In addition, Ding et al. (2013) reported the same tendency in HCC tissues and cell lines. In the human HCC microenvironment, there were upregulated proinflammatory cytokines, which increased HDAC6 expression *via* a proximal nuclear factor κB (NF-κB) binding site on the HDAC6 gene promoter. The above two studies revealed the oncogene role of HDAC6. However, Jung et al. (2012) reported HDAC6 downregulation in patients with HCC, which was associated with poor clinical prognosis, specifically for 5-year overall, disease-free, and recurrence-free survival. Further studies demonstrated that HDAC6 is a tumor suppressor that activates c-Jun NH2-terminal kinase (JNK)–mediated beclin 1–dependent autophagic cell death. Lv et al. (2016) also observed HDAC6 downregulation in HCC; moreover, HDAC6 downregulation was associated with the recurrence rate in patients with HCC undergoing liver transplantation. Further studies suggested that HDAC6 knockdown could contribute to progression and angiogenesis under hypoxia in HCC cells *via* HIF-1α/VEGFA axis. Qiu et al. (2019) reportedthat deacetylase activity of HDAC6 is associated with decreased T_H_17 cell pathogenicity and antitumor immune response. HCC growth was inhibited by the adoptive transfer of HDAC6-deficient T_H_17 cells, which rely on increased IL-17A production by upregulating antitumor cytokine production and CD8^+^ T cell–mediated antitumor responses. HDAC6 could limit T_H_ 17 pathogenicity and antitumor effects by regulating forkhead box O1 (FoxO1). Moreover, deacetylation by HDAC6 of cytosolic FoxO1 at K242 is required for nuclear translocation and stabilization of FoxO1. Nevertheless, the inconsistent reports regarding HDAC6 expression in HCC could be attributed to the studies using different normal liver cell lines and HCC cell lines. The above studies revealed controversial results, which may result from different susceptible pathways, sample size, cell types, and so on; therefore, there is a need for further research on the relationship between HDAC6 and HCC.

Regarding inhibitors, Cho et al. (2013) reported a new series of pyridone-based HDAC inhibitors; among them, (E)-N-hydroxy-3-acrylamide selectively inhibit class I HDAC1 and class II HDAC6 enzymes. Studies have confirmed its metabolic stability in mouse liver microsomal studies; moreover, the growth of various cell lines can be effectively attenuated. Bae et al. (2015) reported that miR-221 suppresses HDAC6; moreover, this suppression was induced by JNK/c-Jun signaling in HCC without effecting normal hepatic cells. Furthermore, miR-221 was independently regulated by cytokine-induced NF-κBp65 upon suppressed HDAC6 expression in HCC cells. Li et al. (2021) observed that long noncoding RNA LINC00624 could disrupt the formation of the HDAC6-TRIM28-ZNF354C transcriptional corepressor complex in HCC, followed by the dissociation of the complex from the promoter of CHD1L and BCL9, which eventually clears the transcription inhibition.

### Cholangiocarcinoma

CCA is a highly fatal tumor, with an increasing worldwide incidence over the past 2 decades. Patients with advanced CCA have poor prognosis, with a median survival of less than 24 months (Francis et al., 2010). Moreover, there could be a relationship between HDAC6 and CCA, which might inform future study.

Boonjaraspinyo et al. (2012) reported HDAC6 mRNA downregulation in CCA tissues compared with adjacent noncancerous tissue. Although there was no relationship between HDAC6 mRNA expression and survival rate, HDAC6 mRNA levels were correlated with CCA staging. Gradilone et al. (2013) reported HDAC6 protein overexpression in CCA cell lines (Hucct-1 and KMCH) compared with normal cholangiocytes. The same tendency was observed in CCA tissue compared with normal tissue; however there was no difference in the mRNA levels. HDAC6 expression was higher in CCA tissues than in normal liver samples. These results suggest the different *in vivo* and *in vitro* effects of the posttranscriptional regulatory pathway of HDAC6. Gradilone et al. suggested that HDAC6 promotes CCA growth by decreasing ciliary expression, which is crucially involved in CCA formation. HDAC6 inhibition with specific shRNA or selective inhibitor tubastatin-A restored ciliary expression, and reduced tumor growth *in vivo* and *in vitro*. Study from the same research group reported the upstream regulator of HDAC6; moreover, miR-433 and miR-22 downregulation induced HDAC6 overexpression and ciliary loss, which suggests their essential role during CCA (Mansini et al., 2018). There remains a lack of related studies on the role of HDAC6 inhibitors in CCA. Therefore, HDAC6 possibly serves as an oncogene in CCA; however, further studies are required for a more confirmed conclusion.

## Conclusions and Perspectives

**FIGURE 3:**
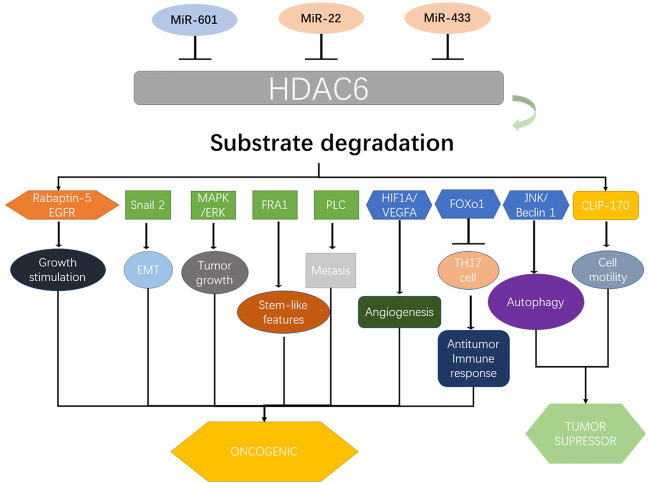
Upstream regulators of HDAC6 and its major downstream targets that contribute to gastrointestinal cancers. HDAC6 coordinates several proteins and pathways including Rabaptin-5, EGFR, Snail 2, MAPK/ERK, FRA1, PLC, HIF1A/VEGFA, FOXO1, JNK/beclin 1, and CLIP-170 and determines their role in various types of gastrointestinal cancers. Various miRNAs including miR-601, miR-22, and miR-433 regulate the expression of HDAC6. MAPK, mitogen-activated protein kinase; ERK, mitogen-activated protein kinase; PLC, phospholipase C; VEGFA, VEGF A; FOXO1, Forkhead box O1; JNK, c-Jun N-terminal kinase; CLIP-170, cytoplasmic linker protein 170.

HDAC6 is a member of HDAC family, which plays an important role in multiple diseases, with its role in gastrointestinal cancer being prominent. Generally, it could be considered as an oncogenic factor in esophageal, colorectal, and pancreatic cancers; however, its role in GC, liver cancer, and CCA remains controversial. Although HDAC6 has been reported in different cancer-related signaling pathways (Figure 3), its exact underlying mechanism remains largely unelucidated. Therefore, further studies are necessary to recover the oncogenic mechanism of HDAC6 in esophageal, colorectal, and pancreatic cancers, as well as its upstream regulation. For GC, liver cancer, and CCA, further studies are needed to further clarify the exact role of HDAC6 in these cancers, or reveal the underlying affecting factors for the controversial results currently. Owing to its effect on the survival and maintenance of the malignant phenotype, HDAC6 could be considered as an idea target for cancer therapy. Although several HDAC6 inhibitors have been reported therapeutic roles in gastrointestinal cancers (Table 1), few have been applied to clinical trials or clinical treatment. Therefore, there is a need to elucidate the relationship between its structure and function, as well as to identify novel HDAC6-selective inhibitors that could benefit clinical strategies for treating gastrointestinal cancers by preclinical cell and animal experiments and clinical trials.

**TABLE 1:** Gastrointestinal cancers and relative HDAC6 inhibitors.

Cancer	HDAC6 inhibitors	Substrate/pathway
Esophageal cancer	SiRNA	α-Tubulin and HSP90
	MiR-601	—
	ACY1215	G2/M arrest and apoptosis via miR-30d/PI3K/AKT/mTOR and ERK pathways
GC	DTBP	Senescence
		Mitotic catastrophe
	TC24	G2/M cell cycle arrest
		HIF-1α and VEGF
CRC	ACY-1215	Caspase-3/poly (ADP ribose) polymerase/Bcl-2/pERK and pPKB
		Programmed death ligand 1
		CHOP and ER stress
	A452 + SAHA	γH2AX and Chk2
	TSA + SAHA	Wnt transcription factor TCF7L2
	Aceroside VIII + A452	Acetylated α-tubulin
	Compound 12	Cy987totoxic effects
	CRA-026440	Acetylated histone and acetylated tubulin
	23bb	α-Tubulin
	Compound 6	Caspase and PARP
	C1A	KRAS
	Tubacin	CD133þ tumor-derived EVs
	MPT0G612	Apoptosis
Liver cancer	(E)-N-hydroxy-3-acry-lamide	—
	miR-221	JNK/c-Jun signaling
Pancreatic cancer	Tubastatin A	—
	CUDC-907	c-Myc and Ki67
	Cefoperazone sodium	—
CCA	—	—

pPKB, phosphorylated protein kinase B.
